# Dendritic polyglycerol nanoparticles show charge dependent bio-distribution in early human placental explants and reduce hCG secretion

**DOI:** 10.1080/17435390.2018.1425496

**Published:** 2018-01-15

**Authors:** Herbert Juch, Liudmila Nikitina, Sabine Reimann, Martin Gauster, Gottfried Dohr, Barbara Obermayer-Pietsch, Denise Hoch, Karin Kornmueller, Rainer Haag

**Affiliations:** a Institute of Cell Biology, Histology and Embryology, Medical University of Graz, Graz, Austria;; b Institute of Chemistry and Biochemistry-Organic Chemistry, Freie Universität Berlin, Berlin, Germany;; c Division of Endocrinology and Diabetology, Medical University of Graz, Graz, Austria;; d Department of Obstetrics and Gynecology, Medical University of Graz, Graz, Austria;; e Institute of Biophysics, Medical University of Graz, Graz, Austria

**Keywords:** Dendritic polyglycerol nanoparticles, early human placenta, hCG, BeWo, primary trophoblasts, nanotoxicology

## Abstract

A thorough understanding of nanoparticle bio-distribution at the feto-maternal interface will be a prerequisite for their diagnostic or therapeutic application in women of childbearing age and for teratologic risk assessment. Therefore, the tissue interaction of biocompatible dendritic polyglycerol nanoparticles (dPG-NPs) with first- trimester human placental explants were analyzed and compared to less sophisticated trophoblast-cell based models. First-trimester human placental explants, BeWo cells and primary trophoblast cells from human term placenta were exposed to fluorescence labeled, ∼5 nm dPG-NPs, with differently charged surfaces, at concentrations of 1 µM and 10 nM, for 6 and 24 h. Accumulation of dPGs was visualized by fluorescence microscopy. To assess the impact of dPG-NP on trophoblast integrity and endocrine function, LDH, and hCG releases were measured. A dose- and charge-dependent accumulation of dPG-NPs was observed at the early placental barrier and in cell lines, with positive dPG-NP-surface causing deposits even in the mesenchymal core of the placental villi. No signs of plasma membrane damage could be detected. After 24 h we observed a significant reduction of hCG secretion in placental explants, without significant changes in trophoblast apoptosis, at low concentrations of charged dPG-NPs. In conclusion, dPG-NP’s surface charge substantially influences their bio-distribution at the feto-maternal interface, with positive charge facilitating trans-trophoblast passage, and in contrast to more artificial models, the first-trimester placental explant culture model reveals potentially hazardous influences of charged dPG-NPs on early placental physiology.

## Introduction

Nanomaterial interaction with human placenta is insufficiently understood. This impedes the development of beneficial nanomaterial-based diagnostics and treatments for pregnant women (Keelan et al. 2015; Harris 2016; Muoth et al. 2016) as well as embryonic risk assessment of human first trimester exposures to nanomaterials. Models commonly used to analyze placental nanomaterial kinetics and tissue compatibility either focused on late human gestation only, like the human placental perfusion model (Grafmueller et al. 2015), or primarily studied human trophoblast-tumor cell interactions with nanomaterials, e.g. based on the BeWo-b30 choriocarcinoma models (Cartwright et al. 2012; Ali et al. 2013; Correia Carreira et al. 2015a; Poulsen et al. 2015). Animal models for the investigation of nanomaterial exposures in early gestation are predominantly based on rats and mice. The results, however, are difficult to translate to the human situation since these models analyze nanomaterial kinetics at the yolk sac–placenta, a structure completely different from the early human placenta (Carney et al. 2004). The guinea pig model is comparatively advantageous because of a series of similarities to human placentation (Grigsby 2016) and has been used to study a NP-based vaccine in pregnancy (Glenn et al. 2015), but early placentation in guinea pigs is also quite different from humans.

However, studies have confirmed hypotheses on parameters relevant for the uptake of nanomaterials in cells and tissues, e.g. dose-, size-, and surface charge-dependency (Oberdorster, Oberdorster, and Oberdorster 2005; Maynard, Warheit, and Philbert 2011), in different placenta models (recently reviewed by Keelan et al. 2015) and the limitations of the approaches have been discussed (Correia Carreira et al. 2015b; Keelan et al. 2015; Nikitina, Dohr, and Juch 2015). For mouse placenta, a charge- and dose-dependency of iron-oxide NP accumulation in the fetus was confirmed (Di Bona et al. 2014). In a recent study, targeted liposomes were shown to deliver cargoes of carboxyfluorescein and insulin-like growth factor 2 into the mouse placenta, without interfering with fetal development; human first trimester and term placental explants showed a similar distribution of these liposomes (King et al. 2016).

The variability of engineered nanomaterials seems to be endless, promising possible solutions for various diagnostic- and treatment-problems in medicine, but also suggesting novel hazards. Dendritic polyglycerol nanoparticles (dPG-NPs) are of special interest, e.g. as drug carriers, since they can be designed in a controlled manner to obtain a defined size and functionalization (Calderon et al. 2010). Determined structure-biocompatibility relationships of dPG-NPs indicate their suitability for a systemic administration (Khandare et al. 2010). The dependency of cellular dPG-NP-uptake on surface charge was described for epithelial cell lines and *in vivo* experiments (Gröger et al. 2013; Pant et al. 2015).

To expand the knowledge in this field, we have employed an established approach to investigate the unique situation at the early human maternal-embryonic interface, the human first- trimester explant culture model (Miller et al. 2005). The objective was to analyze time-, dose-, and charge-dependency of dPG NP distribution at the early human placental barrier. In addition, we studied the impact of dPG-NP exposure on LDH release by explants, BeWo cells, and primary trophoblasts, to assess potential plasma membrane damage and the impact on human chorionic gonadotropin (hCG) secretion, to detect alterations of trophoblast physiology. Moreover, the comparison of results of the different models should improve our understanding of the usability of easier to handle systems as a substitute for placental explant culture.

## Materials and methods

### dPG-NP synthesis and characterization

All chemicals were reagent grade, used without further purification, and purchased from Acros Organics (Geel, Belgium), Sigma-Aldrich (Steinheim, Germany), Fluka (Buchs, Switzerland), Merck KGaA (Darmstadt, Germany), and Deutero (Kastellaun, Germany). Reactions sensitive to moisture or air were carried out under argon atmosphere using anhydrous solvents and flame-dried glassware. Dialysis was conducted in benzoylated cellulose tubing purchased from Sigma-Aldrich (MWCO 2000 g mol^−1^) changing the solvent at least four times over a period of 48 h. Ultrafiltration was conducted in solvent-resistant stirred cells (Millipore, Merck KGaA) with PLAC regenerated cellulose membranes (MWCO 1000 g mol^−1^). SEC was performed with Sephadex™ G-25 superfine (Sigma Aldrich) in distilled water under room temperature and pressure. ^1^H- and ^13^C-NMR spectra were recorded on a Jeol ECX 400 spectrometer (Jeol, Tokyo, Japan) or on a Bruker Biospin Avance 700 spectrometer (Bruker, Billerica, MA, USA). Chemical shifts (δ) were reported in ppm using the deuterated solvent peak as the internal standard (D_2_O: δ (^1^H) = 4.79 ppm; MeOD-d4: δ (^1^H) = 3.31 ppm, δ (^13^C) = 49.00 ppm; CDCl_3_: δ (^1^H) = 7.26 ppm, δ (^13^C) = 77.16 ppm). IR measurements were recorded on a Nicolet Avatar 320 FT-IR equipped with a DTGS detector from 4000 to 650 cm^−1^ and evaluated using the software EZ OMNIC ESP. Wavenumbers υmax were reported in cm^−1^; intensities of the absorption bands were assigned as strong (s), medium (m), and weak (w). Elemental analysis to determine the degree of sulfation was performed on a VARIO EL III instrument (Elementar, Hanau, Germany) using sulfanilic acid as the standard. The average dye incorporation per polymer was calculated by UV–Vis spectra from a 5 µM solution in PBS at pH 7.4 recorded on a LAMBDA 950 UV/Vis/NIR spectrometer (PerkinElmer, Waltham, MA, USA) at 25 °C. DLS and ζ-potential measurements were carried out on a Zetasizer Nano ZS (Malvern Instruments Ltd., Worcestershire, UK) equipped with a 4 mW He–Ne laser (*λ* = 633 nm, NIBS) operating with a 173° scattering angle (backscatter). Hydrodynamic diameters were determined in UV-transparent disposable cuvettes (UltraVette, 8.5 mm, Brand, Wertheim, Germany) at 25 °C. Samples were dissolved at a concentration of 2 mg mL^−1^ in Dulbecco’s PBS (DPBS, without Ca^2+^, Mg^2+^, pH =7.4, PAA, Pasching, Austria). The stated values are the mean of at least 15 independent measurements. ζ-potential measurements were conducted at a concentration of 2 mg mL^−1^ in phosphate buffer (PB, 10 mM, pH 7.4) at 25 °C in folded DTS 1060 capillary cells (Malvern Instruments Ltd.). Malvern Zetasizer Software version 6.12 (Malvern Instruments Ltd.) was used for data evaluation. The stated values and standard deviations are based on the Smoluchowski model and are the mean of at least five independent measurements with 15 scans each.

**Figure 1.**
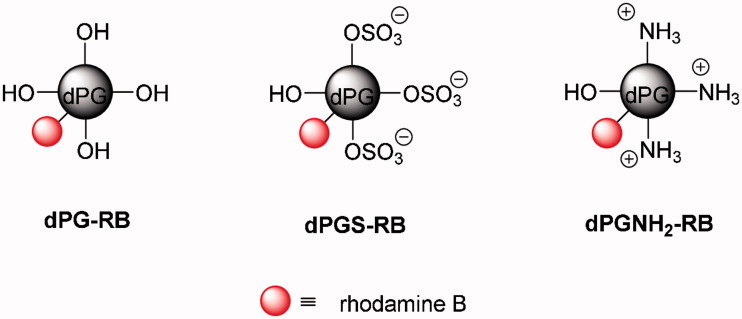
Schematic illustration of rhodamine B labeled dendritic polyglycerol (dPG-RB), dPG sulfate (dPGS-RB), and dPG amine (dPGNH_2_-RB). Counter ions are not shown for clarity reasons.

In order to monitor the charge dependent uptake of nanoparticles by the human placenta, rhodamine B (RB) conjugates of neutral, anionic, and cationic dPG-NPs were synthesized, using a dPG scaffold with a molecular weight of Mn = 6000 g mol^−1^, a polydispersity index (PDI) of ≍1.6, and a degree of branching of 61%. Esterification of dPG with RB using N-hydroxysuccinimide (NHS) and N,N′-dicyclohexylcarbodiimide (DCC) according to a published procedure (Gröger et al. 2014) gave neutral dPG-RB (Figure 1, Supplementary Figure S1) with a dye to polymer ratio of 0.06 (Table 1). The synthesis of anionic RB labeled dPGS (dPGS-RB 5), later referred to as ‘negatively charged dPG-NPs’, was accomplished by partial mesylation and azidation of the neutral polymer, and subsequent sulfation of remaining hydroxyl groups with SO_3_ pyridine complex, followed by the copper-catalyzed click coupling of alkyne functionalized RB to dPGS azide as previously published (Figure 1, Supplementary Figure S2, Gröger et al. 2014). The product was obtained with a dye incorporation of 0.25 and a degree of sulfation of 67% as determined by combustion analysis (Table 1). Cationic dPGNH_2_-RB, later referred to as ‘positively charged dPG-NPs’, was prepared with 0.6 dyes per polymer (Table 1) by mesylation, azidation, and reduction of residual azide groups (Roller, Zhou, and Haag 2005) followed by the conjugation of RB isothiocyanate to dPG amine (6) (Figure 1, Supplementary Figure S2). Derivatives were synthesized with hydrodynamic diameters (D_h_, volume distribution) between 5 and 7 nm as found by dynamic light scattering (DLS) measurements in PBS (pH 7.4) and molecular weights of 6 and 12.1 kDa as calculated from the respective degree of functionalization (dF) (Table 1). Surfaces charges were determined by ζ-potential measurements in phosphate buffer (PB, pH 7.4) with dPGNH_2_-RB showing a value of +12 mV, dPGS-RB of −28 mV and dPG of 0 mV, which are in accordance to previously reported data of non-dye labeled compounds (Weinhart, Becherer, and Haag 2011; Weinhart et al. 2011; Mehrabadi et al. 2015).

**Table 1.** Characterization of rhodamine B labeled dPG nanoparticles.

Polymer	M_n_^a^ (g mol^−1^)	NF^b^	dF^c^	PDI^d^	d_h _±SD^e^ (nm)	ζ-potential ± SD^f^ (mV)	D/P^g^
2 dPG-RB	6000	81	–	0.34	5.3 ± 0.7	0	0.06
5 dPGS-RB	12100	54	67	0.28	6.9 ± 1.1	−27.9 ± 3.3	0.25
7 dPGNH_2_-RB	6000	73	90	0.37	5.6 ± 0.6	+12.1 ± 1.6	0.6

a Number average molecular weight calculated from the dF.

b Number of functional groups per polymer.

c Degree of functionalization determined by ^1^H-NMR or elemental analysis.

d Polydispersity index (DLS).

e Hydrodynamic diameter (mean ± standard deviation (SD)) by DLS in PBS (pH 7.4) from the size distribution by volume.

f ζ-potential (mean ± SD) in PBS (pH 7.4).

g Dye to polymer ratio determined by UV–Vis spectroscopy at 25 °C from a 5 µM solution in PBS.

NanoSightLM14 (Malvern Instruments Ltd.) was used for nanoparticle tracking analysis (NTA) and particle size determination in culture medium; laser wavelength: 532 nm, filter for RB-fluorescence detection: 565 nm. Measurements were done at 25 °C, image capture: CMOS camera, 25 frames per second, capture time 3 × 60 s, with and without filter. NTA version 3.2 Dev Build 3.2.16 software (Malvern Instruments Ltd.) was used for data analysis.

### Cell and tissue culture

The study was approved by the ethic institutional review board of the Medical University Graz (20-261 ex 08/09). First trimester placentas (*n* = 15, mean gestational age = 8.5 ± 1.7) were obtained from elective terminations of pregnancies (ETOP, not Mifepristone-induced) and term placentas (*n* = 3) were taken after cesarean section of non-complicated pregnancies. BeWo cells were obtained from ACC (Sigma-Aldrich). For experiments with first trimester placental explants and BeWo cells DMEM/F-12 (1:1), (Gibco^®^, Life Technologies™, Thermo Fisher Scientific, Vienna, Austria) containing 10% fetal calf serum (FCS, Life Technologies™), 2 mM Glutamin (L-Glutamine 200 mM (100X), Gibco^®^), and 1X antibiotic–antimycotic solution (Anti–Anti (100X), Gibco^®^) was used. For primary cytotrophoblast cell culture, DMEM-HG (Gibco^®^) containing 10% FCS and 1X antibiotic–antimycotic solution was applied. dPG-NPs were diluted in complete culture medium to concentrations of 1 µM and 10 nM. Cells and explants were exposed to dPG-NPs for 6 and 24 h. Placental explants from first trimester placentas were prepared as described by Miller et al. (2005), and incubated overnight in the *ex vivo* closed incubation system (BioSpherix Ltd., Parish, NY, USA) under hypoxic conditions (2.5% O_2_, 5% CO_2_) followed by dPG-NP exposure in 96-well plates (Thermo Fisher Scientific) for 6 h and in 24 well plates for 24 h, on the bottom of the well, without matrix-coating, one explant per well. Explants were then fixed in 4% PFA (Merck KGaA) and embedded in paraffin (Tissue-Tek^®^ VIP, Sysmex, Vienna, Austria) for subsequent histological processing.

BeWo cells were cultivated for 2 d on glass coverslips in the six-well plates with a density of 2 × 10^5^ cells per well (for microscopy) and in 96-well plates with a density of 1 × 10^4^ cells per well (for toxicity tests) and afterwards exposed to dGP-NPs at concentrations 1 µM and 10 nM.

According to the method described by Petroff et al. (2006), primary trophoblast cells were isolated from term placentas and seeded onto Collagen Typ I coated, 4-well Culture Slides (BD Biosciences GmbH, Vienna, Austria) with a density of 0.5 × 10^6^ per chamber (for microscopy) and in 96-well plates with a density of 1 × 10^5^ per well (for toxicity tests) for 2 d, followed by dPG-NP exposure at concentrations of 1 µM and 10 nM.

### Fluorescence and confocal microscopy

Placental explants of 5 µm sections were prepared on adhesive glass slides (Superfrost Plus™, Thermo Fisher Scientific) deparaffinized, counterstained with DAPI (Vectashield^®^ Mounting Medium for Fluorescence with DAPI, Vector Laboratories, Szabo-Scandic GmbH, Vienna, Austria) and analyzed, using a fluorescence microscopy system Leica TCS SP2 (Leica Microsystems GmbH, Vienna, Austria) equipped with a digital camera (AxioCam HRc, Carl Zeiss GmbH, Vienna, Austria).

Biotinylated dPG-NPs were detected, using a Streptavidin-Peroxidase-AEC detection system (Thermo Fisher Scientific).

After exposure to dPG-NPs, BeWo cells from coverslips and primary trophoblast cells from chamber slides were washed three times using HBSS (Gibco^®^), fixed with 4% PFA for 20 min and counterstained with DAPI for evaluation on the confocal microscope. Confocal microscopy was performed to distinguish possible NP deposition on the outer cell surface from intracellular dPG-NP deposits by choosing the optical sections at the estimated equatorial level of the nuclei for analysis. Amira software version 3.1 (TGS Template Graphics Software Inc., Bedford, UK) was used for 3D reconstructions from Z-stacks.

### Analysis of cytotoxicity and endocrine function

Supernatants from cells and placental explants were frozen immediately at −80 °C after centrifuging for 5 min at 1000 rcf (Heraeus™ Megafuge™ 40R, Thermo Fisher Scientific). Control measurements were performed to exclude assay interaction with FCS or nanoparticles. Unexposed cells and tissues were used as negative controls, Triton^®^X100 (Merck KGaA; 1% vol. diluted in medium) exposure was used to generate positive controls.

Lactate dehydrogenase (LDH) release was measured using a LDH Cytotoxicity Detection Kit (Takara Bio Europe Inc., Saint-Germain-en-Laye, France), following the manufacturer’s instructions.

### TUNEL assay

TUNEL technology, as described by the manufacturer (Roche Applied Science, Sigma-Aldrich), was used to assess apoptosis. The trophoblast layers were identified by E-Cadherin immunostaining, using monoclonal anti-E-Cadherin (clone SPM471, Thermo Fischer Scientific) and Alexa Fluor 633 goat anti-mouse IgG (Molecular Probes^®^, Thermo Fisher Scientific). Images were captured, using a Zeiss LSM 510 META Axiovert 200M Zeiss confocal system (Carl Zeiss) with a 40× Plan-Neofluar 1.3 DIC oil immersion objective. A mean of 1249 (range: 958–1755) trophoblast-nuclei was evaluated in ten randomly selected areas for each treatment.

### hCG measurement

The concentrations of hCG were determined in diluted culture supernatants using the IMMULITE 2000 immunoassay system (Siemens Healthcare Diagnostics GmbH, Vienna, Austria). dPG-NPs (1 µM) were added to control samples after culture to detect a potential direct interference of the dPG-NPs with the assay.

### Data analysis

All measurements were performed in duplicates, quantitative data are displayed as mean values ± standard error of mean (SEM) of three independent placental explant culture experiments, three independent primary trophoblast cultures, and three independent BeWo cell cultures. ANOVA or Kruskal–Wallis test was performed, using Graph pad prism 7 (GraphPad Software, La Jolla, CA, USA) for Windows, to evaluate the data and Bonferroni/Dunn’s post hoc test was employed to analyze the statistical significance of differences between exposures and controls. *p* values <0.05 were considered significant.

## Results

### Localization of dPG-NP deposits in trophoblasts and placental explants

**Figure 2.**
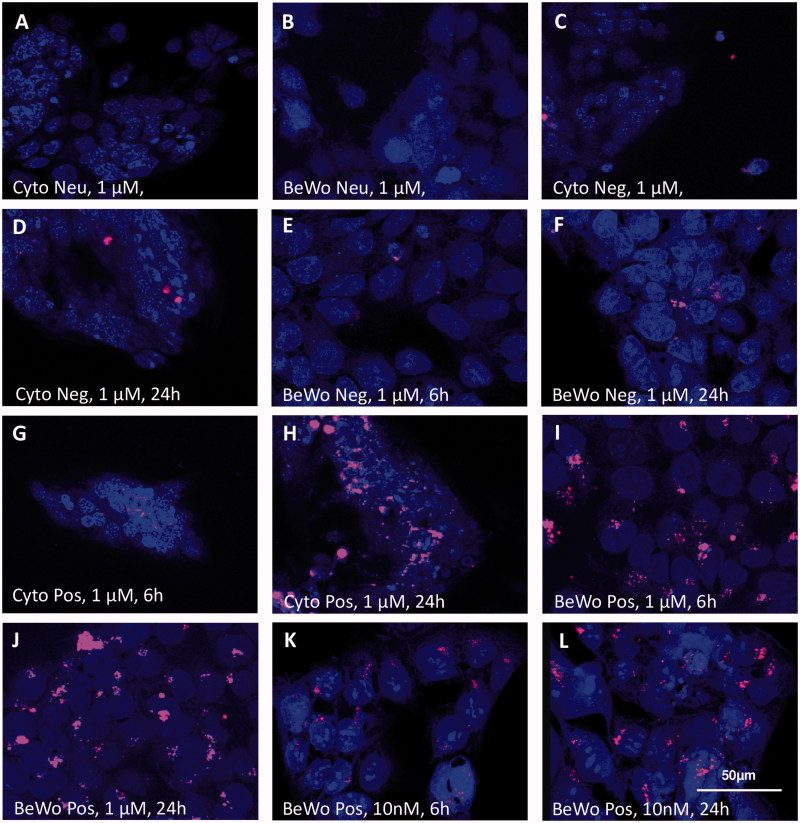
Localization of differently charged (neutral (Neu; A,B), negative (Neg; C–F), positive (Pos; G–L)), labeled dPG-NPs (pink) in exposed primary cytotrophoblasts (A–D,G,H), and BeWo cells (B,E,F,I–L). Exposure concentrations: 1 µM (A–J) and 10 nM (K,L)), exposure time: 6 h (C,E,G,I,K), and 24 h (A,B,D,F,H,J,L). Nuclear staining: DAPI (blue). Confocal images, 400x. Patchy perinuclear and nuclear localization was seen after 6 h of exposure to 1 µM neg. dPG-NPs (C,E) and pos. dPG-NPs (G,I), increasing after 24 h. Notably, in BeWo cells pos. dPG-NP deposits were also detected after exposing them to a 10 nM concentration for 6 h (K) and 24 h (L). No neutral dPG-NP deposits were detected in both cell types (A,B).

**Figure 3.**
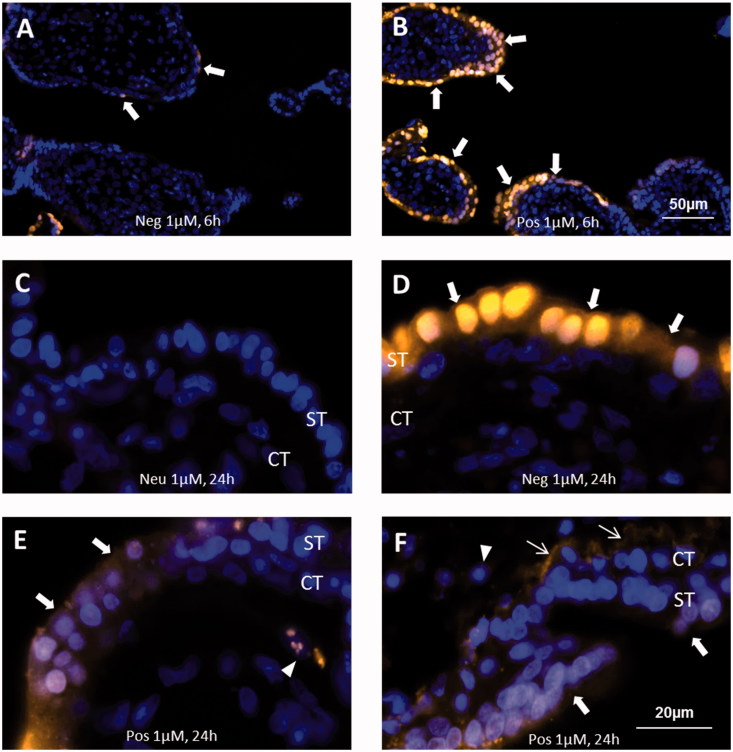
Localization of differently charged (neutral (Neu; C), negative (Neg; A,D), positive (Pos; B, E–F)), labeled dPG-NPs (orange), in exposed placental explants. Exposure concentration: 1 µM, exposure-time: 6 h (A,B) and 24 h (C–F). Nuclear staining: DAPI (blue). Fluorescence microscopy images, 200× (A,B), 1000× (C–F). Local staining of cytoplasm and nuclei in syncytiotrophoblast (ST, arrows) was detected after 6 h of exposure to neg. and pos. dPG-NPs (A,B), increasing after 24 h. Notably, pos. dPG-NP deposits, were localized at the basal membrane (thin arrows, F) close to the cytotrophoblast cell layer (CT) and in some cells of mesenchymal core (short arrowheads) (E,F). No signal was observed for neu. dPG-NPs (C).

Microscopy analysis revealed a charge-, exposure time-, and dose-dependent dPG-NP accumulation in both, trophoblasts and placental tissues (Figures 2 and 3). Deposits of negatively and positively charged dPG-NPs were detectable in explants and cells after 6 h of incubation, but no visible neutral dPG-NP deposits appeared, even after 24 h of exposure (Figure 2(A,B); Figure 3(C)). Biotin-labeled neutral dPG-NPs were also undetectable in explants after 1 µM exposure for 24 h (Supplementary Figure 3). Positively charged dPG-NPs (pos. dPG-NPs) showed an increased cellular accumulation compared to negatively charged dPG-NPs (neg. dPG-NPs) (Figure 2(C–F,G–L)); Figure 3(A,B)), but considering the dye functionalization (D/P values in Table 1), this apparent difference might be due to a better detectability of pos. dPG-NPs. However, the comparatively more intensive fluorescence signal in the syncytium in Figure 3(D) indeed indicates a higher amount of neg. dPG-NPs in the syncytium compared to pos. dPG-NPs (Figure 3(E,F)), after 24 h of exposure.

Neg. dPG-NP deposits remained clearly restricted to the syncytium, even after 24 h of exposure (Figure 3(D)). Notably, 24 h exposure to pos. dPG-NPs resulted in deposits also in the cytotrophoblast layer, at the basal membrane and even in cells of the mesenchymal core (Figure 3(E,F)).

dPG-NP deposits in cells and tissues were not evenly distributed, but did show a patchy accumulation pattern with a preference of the perinuclear area of cells (Figure 2(C–L); Figure 3(A,B,E,F); Supplementary Figure S5). In areas of very intensive dPG-NP accumulation, a more homogeneous staining of the cytoplasm occurred. As seen in the confocal images, intra-nuclear deposits in trophoblast cells were rather an exception. Nuclear uptake is difficult to assess in paraffin sections, since superposition of perinuclear accumulations may appear as intra-nuclear deposits, which cannot be ruled out after 24 h of exposure (Figure 3(D–F)).

Interestingly, only BeWo cells showed cytoplasmic and nuclear deposits of pos. dPG-NPs after 10 nM exposure (Figure 2(K,L)), although the degree of charged dPG-NP accumulation after 1 µM exposures did not appear to be substantially higher in BeWo cells (Figure 2(I,J)) compared to primary cytotrophoblasts (Figure 2(G,H)) and explants (Figure 3(B,E,F)).

### Toxicity and endocrine function

**Figure 4.**
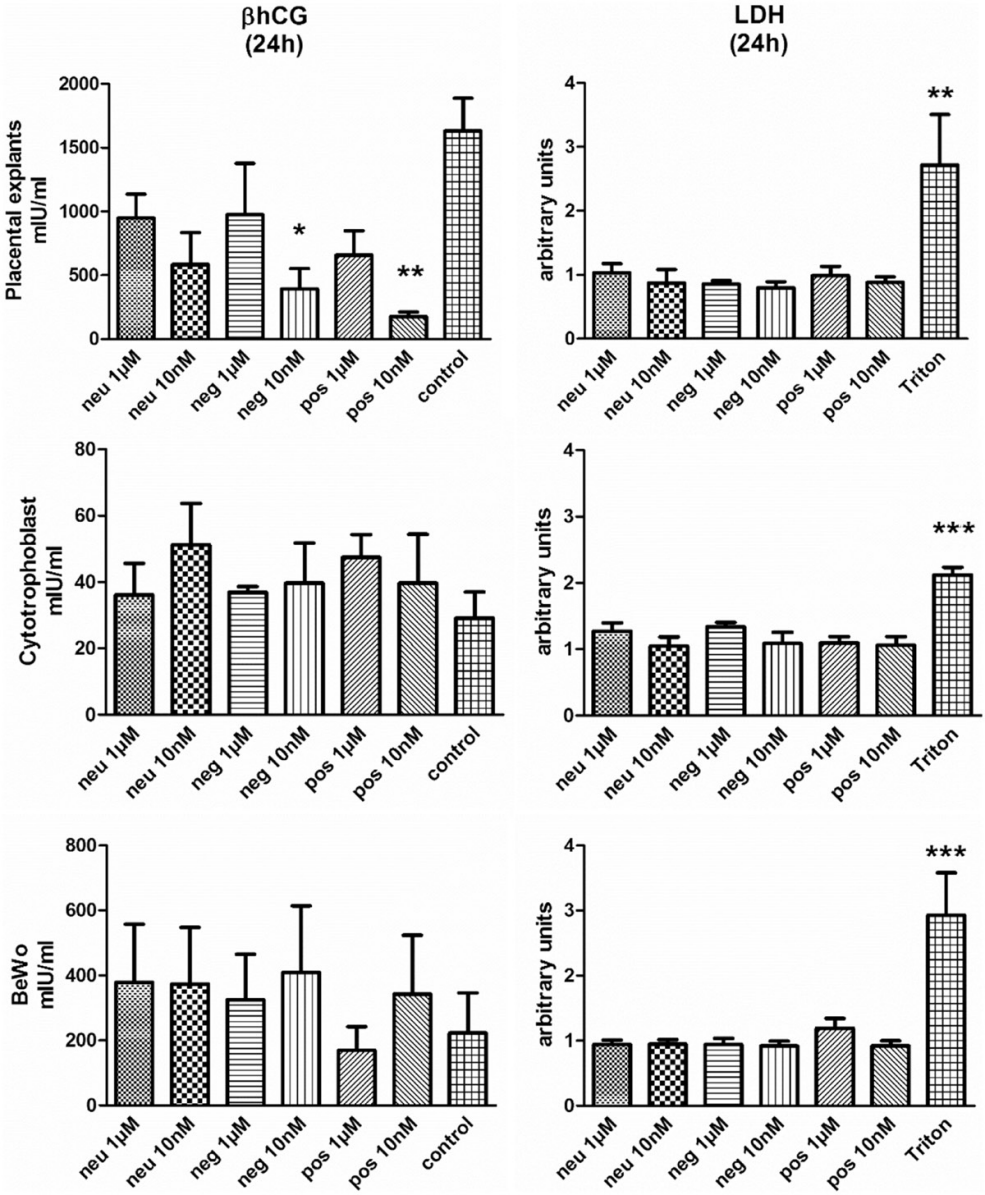
LDH- and hCG-levels in culture supernatants of placental explants, BeWo cells, and primary cytotrophoblast cells, exposed to neutral (Neu), positively (Pos), and negatively charged (Neg) dPG-NPs for 24 h, at concentrations of 1 µM and 10 nM and to 1% Triton^®^X100. LDH data are shown in relative units as ratio to control values, while hCG data are presented in mU mL^−1^. *(*p* < 0.05), **(*p* < 0.01), and ***(*p* < 0.001) indicate statistically significant differences.

Evaluation of LDH release did not reveal dPG-NP exposure to have any significant influence on LDH activity in supernatants from cells and explants (Figure 4 and Supplementary Figure S4), while exposure to Triton^®^X100 clearly increased LDH release. dPG-NP exposure of BeWo and primary trophoblast cells did not significantly change their hCG release (with a tendency toward increase), however, 10 nM pos. dPG-NP and neg. dPG-NP exposure for 24 h significantly decreased hCG release in placental explants, while no dPG-NPs deposits could be detected. A similar, but insignificant trend was observed after 6 h of incubation and for neutral dPG-NPs after 24 h (Figure 4 and Supplementary Figure S4). TUNEL analysis did not show significant differences in trophoblast apoptosis rates after 24 h compared to control (Supplementary Figure S6).

Adding dPG-NPs to supernatants from unexposed control samples after culture did not alter the results of hCG measurements (data not shown).

### NTA in culture medium

Adding dPG-NPs (1 µM or less) to the culture medium did not change the size distribution of detectable particles. No formation of RB-fluorescent agglomerates or aggregates above the detection limit of 30–40 nm could be observed when using the 565 nm filter (Supplementary Figure S7).

## Discussion

This is to our knowledge the first study describing charge dependent soft matter NP interaction with first-trimester human placenta in comparison to BeWo and primary trophoblast cells.

Dose-, charge-, and time-dependent intracellular accumulation of dPG-NPs are in agreement with previous studies using highly similar particles in non-trophoblast cells (Gröger et al. 2013), suggesting a surface charge to be necessary for cellular uptake. Neg. dPG-NPs show a distribution restricted to the syncytiotrophoblast in early placenta, similar to maternal immunoglobulin G (IgG) distribution in early placenta (Bright, Ockleford, and Anwar 1994), indicating possible similarities between the accumulation processes of ‘natural’ NPs like IgG and neg. dPG-NPs. Iron oxide NP studies in mice (Di Bona et al. 2014) also revealed a charge dependency of placental transfer in rodents. The affinity of positively charged particles to negatively charged loci of the plasma membrane could be responsible for an increased uptake into cells (Zhang et al. 2015). However, the actual charge of NPs in the medium, containing 10% FCS, considering an expectable interaction with charged (e.g. protein) constituents of the medium, remains to be elucidated. In this context, the phenomenon of a protein-corona has been repeatedly discussed (Nel et al. 2009). Notably, for dPG-NPs of ∼5 nm in diameter (about the size of albumin), a dPG-NP-mediated formation of a ‘protein cloud’ in such complex biofluids should be expected, including loosely attached, probably rapidly exchanging layers of biomolecules (Docter et al. 2015).

While neg. dPG-NPs appear to accumulate restricted to the syncytium, pos. dPG-NPs seem to reach the mesenchymal core of the villi and might therefore be less abundant in the syncytium after 24 h of exposure. This remarkable difference might be caused by a preferable adherence of positively charged NPs to transcytosis pathways in early placenta, e.g. to flotillins (Walton et al. 2013).

The patchy distribution of NP accumulations inside cells and the trophoblast layers suggests an active transport of dPG-NPs into the cells and, at least initially, a regulated intracellular deposition rather than a passive diffusion. Membrane defects allowing a passive diffusion of ∼5 nm particles should have a substantial impact on viability, comparable to perforins from cytotoxic T-cells, and cause considerable necrosis and LDH release at least after 24 h of persistence. An inhomogeneity of surface exposure due to dPG-NP-related sedimentation and agglomeration cannot be ruled out. Nevertheless, the density of the dPG-NPs is lower than the density of albumin in the medium, and the low concentration of 1 nM (compared to ∼50 nM for albumin) should also facilitate a homogeneous distribution, comparable to the distribution of the FCS-proteins in the culture medium.

Indeed, an endocytosis-mediated uptake mechanism has been suggested for macromolecules or aggregates in general and NPs in particular (Hillaireau and Couvreur 2009). Assuming an active uptake, our data suggest a functional specialization within the syncytium. The regionally restricted dPG-NP accumulation, especially after 6 h, may be due to a functional heterogeneity in terms of endocytosis activity and particle deposition in first trimester syncytium, which is generally considered to be homogeneous, looking at organelle distribution, in contrast to the syncytium in the second and third trimester (Benirschke and Kaufmann 2012).

However, relatively little is known about the intracellular fate and function of nanoparticles (Deng and Gao 2016). Particularly noteworthy is their possible impact on lysosomal function, which is a key to using NPs as a delivery device, as well as a potential cause of nanomaterial-mediated toxicity. The proton sponge hypothesis posits that unsaturated amines on the surface are capable of sequestering protons, thus keeping the pump of late endosome going and thereby causing late endosomal/lysosomal swelling and rupture, leading to particle release into the cytoplasm (Deng and Gao 2016). This mechanism could be employed by the pos. dPG-NPs . It also remains to be elucidated in which compartments or organelles the dPG-NP accumulations exactly occur and to which extend the particles are expelled by the trophoblast. dPG-NPs tagged to be detectable in electron microscopy could resolve such questions in further studies.

Neither in trophoblasts nor in placental tissue exposed to dPG-NPs did we find evidence for cell membrane damage and LDH-release, compared to controls. This is in line with previous observations, showing no acute toxicity of dPG-NPs (Gröger et al. 2013). Interestingly, we found a significant time-dependent and a charge-dependent effect of dPG-NPs (10 nM) on hCG secretion in placental explants. The physiologic factors regulating hCG secretion *in vivo* are still not fully understood. Much of what is known about factors that stimulate (FGF, calcium, glucocorticoids, phorbol esters, dynorphin and leptin) or inhibit (TGF-β, follistatin and progesterone) hCG-synthesis and secretion has been learned from *in vitro* studies, which are difficult to extrapolate to the *in vivo* situation (Barnea, Shurtz-Swirski, and Kaplan 1992). There is strong evidence for cytotrophoblast and syncytiotrophoblast derived Gonadotropin-releasing hormone (GnRH, identical to hypothalamic GnRH), playing an important role in hCG secretion, and GnRH antagonists have been shown to decrease the basal hCG production. In addition, hCG has been found to bind to the LH/hCG receptor (LH/hCGR) and hCG mRNA expression has been suggested to be autoregulated via LH/hCGR, present on the trophoblast cells (Belisle et al. 1992). Placental hCG secretion is mediated by the secretory granule-exocytosis pathway (Morrish, Marusyk, and Siy 1987) and plays an important role in gestation. Besides its endocrine function, hCG acts via autocrine and paracrine pathways on cytotrophoblast differentiation, uterine angiogenesis, placental immunology, uterine growth, quiescence of uterine muscle contractions and even the growth and development of fetal organs and the umbilical cord (summarized by Cole 2010; Fournier, Guibourdenche, and Evain-Brion 2015).

Based on the available data, two explanations for our observations can be hypothesized: an uptake-independent dPG-NP-interaction with the apical membrane of the syncytium (1) via an undefined specific receptor, causing interference with the above-mentioned regulatory pathways or (2) by unspecific blocking of exocytosis at the syncytiotrophoblast membrane.

Interestingly, the effect reached significance at 10 nM concentrations of dPG-NPs. This phenomenon may be explained by dPG-NPs’ concentration-dependent aggregation and agglomeration tendency or a concentration-dependent binding to different biomolecules in the culture medium. If a receptor-mediated effect is assumed, membrane receptor desensitization or downregulation at higher concentrations of dPG-NPs might be causative (Bourne and von Zastrow 2001).

Noteworthy, the effect only occurred in placental explants but not in the other models. This pinpoints the necessity of complex models, to understand potential adverse effects of NPs at the feto-maternal interface, and the mandatory wariness when using only trophoblast cell lines, as a model of the placental barrier (Juch et al. 2013; Nikitina, Dohr, and Juch 2015). The presence of the original apical syncytial membrane of early placenta, containing a unique selection of specific membrane glycoproteins and lipids, might be required for the observed response. As previously suggested (Kao et al. 1988), Muoth et al. also clearly demonstrated the high relevance of the extracellular matrix for endocrine function and hCG secretion of trophoblast cells, and showed a NP-mediated reduction of hCG release in a 3D BeWo/placental fibroblast co-culture micro tissue model (Muoth et al. 2016).

In addition, we observed differences in the accumulation of dPG-NPs between explants and the cell culture models. Explants and primary cells did not show a deposition of pos. dPG-NPs at a concentration of 10 nM, while BeWo cells did. This indicates either a difference in the uptake mechanism or in the excretion pathways between the choriocarcinoma cells and non-malignant trophoblast.

### Strengths and limitations

An important strength of this study is the use of human first-trimester explants cultures. Insight into charge-dependent dPG-NP interaction with this very specific barrier tissue is essential for understanding possible influences of soft matter NP exposures in pregnancy on human embryonic development. We also used rather low (1 µM, 10 nM) dPG-NP concentrations to simulate a realistic exposure scenario; dose recommendations for a common anti-TNFα therapy (2.5–10 mg/kg body weight) result in plasma concentrations of the therapeutic protein-NPs (monoclonal antibodies) in about this range. Comparing this model to other placenta models contributed to an understanding of important differences in dPG-NP interaction, e.g. concerning the dose-dependent accumulation of dPG-NPs within different cells and tissues, or their influence on placental hormone secretion.

However, our study has some inevitable limitations. First of all, the validity of the first-trimester explant culture model for studying dPG-NP uptake and dPG-NP-mediated effects in early human pregnancy has not been verified so far. Particularly the dPG-NP surrounding in the culture medium is still non-physiologic compared to the situation in utero, e.g. in terms of protein composition and concentrations. Especially the lack of clotting factors and mucin components, which is expected to be relevant for particle agglomeration and aggregation, but also the absence of the intervillous space-specific pulsatile flow, might have a significant impact on dPG-NP uptake (Juch et al. 2013). Additionally, in contrast to the term placenta perfusion model, where intervillous space and fetal vessels are fully separated by the placental barrier, arteries, and veins of stem villi, opened at the cutting site, allow diffusion of culture media components into the fetal vessels. Therefore, NP deposits at the cutting site of the explant and in the fetal vessels have to be interpreted with caution. For this reason, we focused our analysis primarily on the distal parts of the explants (immature intermediate villi and mesenchymal villi). Furthermore, we could not locate dPG-NP- deposits in any of the vessels in our explants after 24 h of dPG-NP incubation.

Consequently, although working directly with tissue representing the early human placental barrier, deductions drawn from our results for the *in vivo*-situation will remain hypothetical until future accidental or inadvertent dPG-NP exposures in pregnancy will allow validation by analyzing maternal hCG levels or particle distribution in early placenta from spontaneous abortions or elective terminations of pregnancy.

The limited sensitivity of the visualization systems, not allowing the detection of single 5 nm particles, precludes ruling out an uptake into cells and tissues. Anyway, the detection of the dPG-NPs was additionally limited by a biochemically restricted D/P ratio <1 (Table 1). While the D/P ratios of negatively- and positively charged dPG-NPs do not differ substantially, the neutral dPG-NPs are clearly less intensively labeled than the charged dPG-NPs. This prevents us from being able to determine quantitative differences in dPG-NP-uptake between charged and uncharged dPG-NPs based on fluorescence intensity and might explain why we could not observe an uptake of neutral dPG-NPs in cells and tissues. However, using neutral dPG-NPs labeled with biotin instead of rhodamine, with a functionalization of ∼2% corresponding to a biotin/particle ratio of ∼1.6, and a highly sensitive streptavidin-peroxidase based detection system, we could not detect any neutral dPG-NP accumulation in explants either (Supplementary Figure S3).

Purification of dPG-NPs after interaction with diluted FCS was impossible and direct investigations of at least the analytically accessible biomolecule- and protein-attachments were not feasible. Investigating a possible dPG-NP aggregation in culture medium via NTA was technically limited to aggregates of a hydrodynamic diameter >40 nm, therefore, we cannot exclude a formation of smaller dPG-NP aggregates in our culture medium.

In addition, dye leakage from the dPG-NPs due to the cleavage of the ester bond cannot be fully excluded. Under physiological conditions, however, rhodamine dyes usually show a high stability (see e.g. Gonçalves 2009). Of note, esters can be cleaved under acidic conditions as well as upon treatment with enzymes like esterase. We do not know to which extend these phenomena occurred in our physiologic culture media, but if there was a rhodamine leakage causing significantly unspecific tissue staining, we would rather expect a qualitatively similar staining pattern for neutral, negative and positive dPG-NP exposures.

One might speculate in this context that prolonged persistence of large amounts of dPG-NPs in endosomes and lysosomes would lead to a release of rhodamine, due to the acidic conditions and possibly catalyzed by lysosomal enzymes. Leakage of free rhodamine from the lysosomes could lead to a more homogeneous cytoplasmic and nuclear staining as observed in Figure 3(D).

The sensitivity of LDH-measurements for plasma membrane damage in syncytiotrophoblast as a proxy for compromised viability of the villi may be limited. We cannot exclude that the observed significant reduction in hCG secretion is a consequence of an NP- mediated general impairment of trophoblast metabolism or the disturbance of the unique cytotrophoblast-syncytiotrophoblast interaction (e.g. fusion) occurring in explants. However, a TUNEL-test did not indicate a significant influence of dPG-NP exposure on the percentage of apoptotic trophoblast nuclei after 24 h exposure, compared to control. An observable trend toward an increase of TUNEL positive nuclei in villi exposed to dPG-NPs at 10 nM might indicate that a thorough investigation into the complex apoptosis- phenomena, physiologically occurring in placental trophoblast bilayers (Huppertz 2010) would reveal significant effects, but this should be investigated in detail in future studies.

## Conclusion

Based on LDH measurements, dPG-NPs show charge-, time-, and dose-dependent accumulation in early placental explants, BeWo cells and primary cytotrophoblast cells without acute toxic effects. A positive charge seems to facilitate placental transfer into the mesenchymal core of the villi, and thus potentially into the fetal circulation. On the one hand, this might be an important aspect for developing nano-based therapeutics or diagnostics for the fetus. On the other hand, this observation also contributes to teratologic risk assessment of nanomaterial, where positive charge would suggest a higher teratogenic potential. Additionally, the observation of a charge-dependent effect on secretion of hCG in placental explants, especially at very low concentrations, reminds us that we also have to expect unpredictable effects of NPs *in vivo*, e.g. a reduction of the hCG secretion. Our observation suggests that hCG levels in the first trimester could be considered as an additional functional parameter in studies on particle effects in pregnant women, e.g. in context with air pollution (van den Hooven et al. 2012).

Various adverse pregnancy outcomes, such as pregnancy loss, intrauterine growth restriction, pre-eclampsia or preterm birth, as well as a disturbance of the maternal thyroid regulation in early gestation (Korevaar et al. 2016) could possibly result from a reduced hCG secretion and should be considered in teratologic studies on particle exposure.

The fact that the latter phenomenon was found in early placental explants, but not in simple trophoblast monolayer cultures, pinpoints the importance of choosing proper models for investigating NP effects on the feto-maternal interface.

## Supplementary Material

H._JUCH_ET_AL_Supplementary_content.zip
