# Supplementary material for: Dendritic polyglycerol nanoparticles show charge dependent bio-distribution in early human placental explants and reduce hCG secretion
Source: Nanotoxicology. 2018 Jan 15;12(2):90–103. doi: 10.1080/17435390.2018.1425496 (PMC5815307; doi:10.1080/17435390.2018.1425496)
Supplement: H._JUCH_ET_AL_Supplementary_content.zip [file INAN_A_1425496_SM7224.zip › H. JUCH ET AL_Supplementary content/Supplementary_Figures.pdf]

Supplementary figure S1

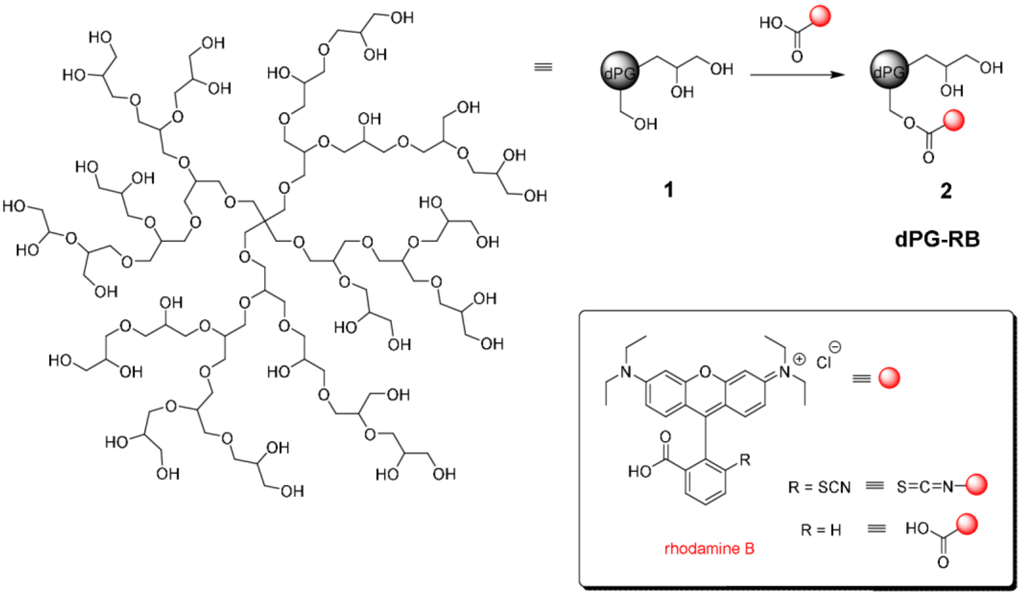

Idealized illustration of the dendritic polyglycerol (dPG) scaffold 1 (Mn = 6000 g mol-1), chemical structure of rhodamine B (RB), and synthetic pathway for RB labeled dPG (dPG-RB 2) via esterification.

Supplementary figure S2

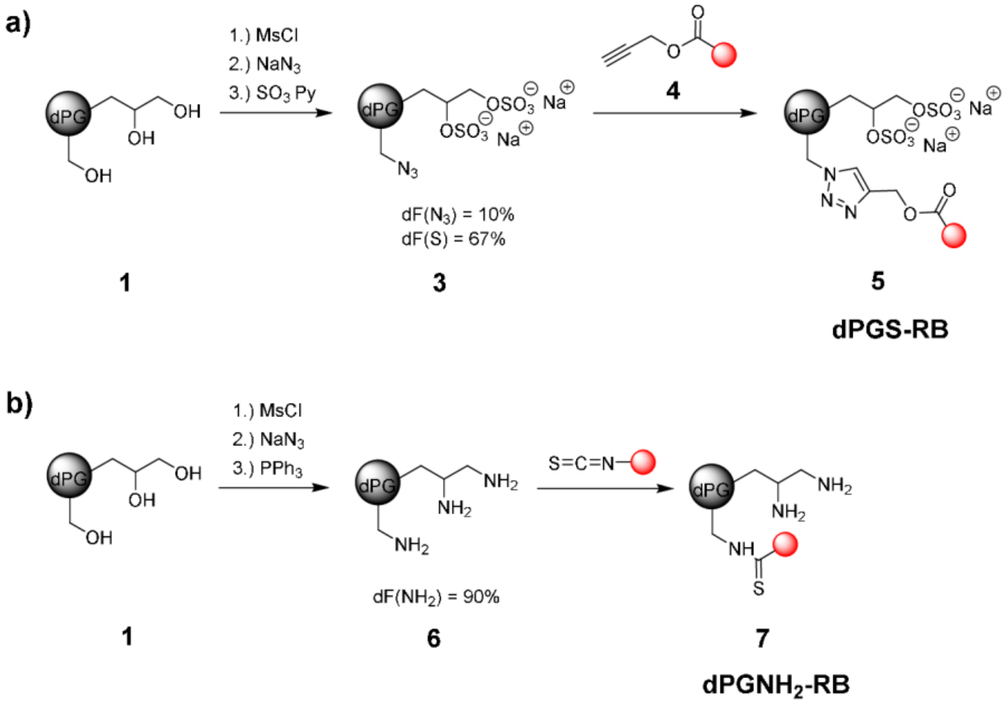

a) Preparation of dPGS-RB (**5**) by partial mesylation, azidation, sulfation and click reaction with alkyne functionalized RB (**4**) b) Synthetic pathway for dPGNH$_2$-RB (**7**) via partial mesylation, azidation, reduction, and coupling of RB isothiocyanate.

Supplementary figure S3

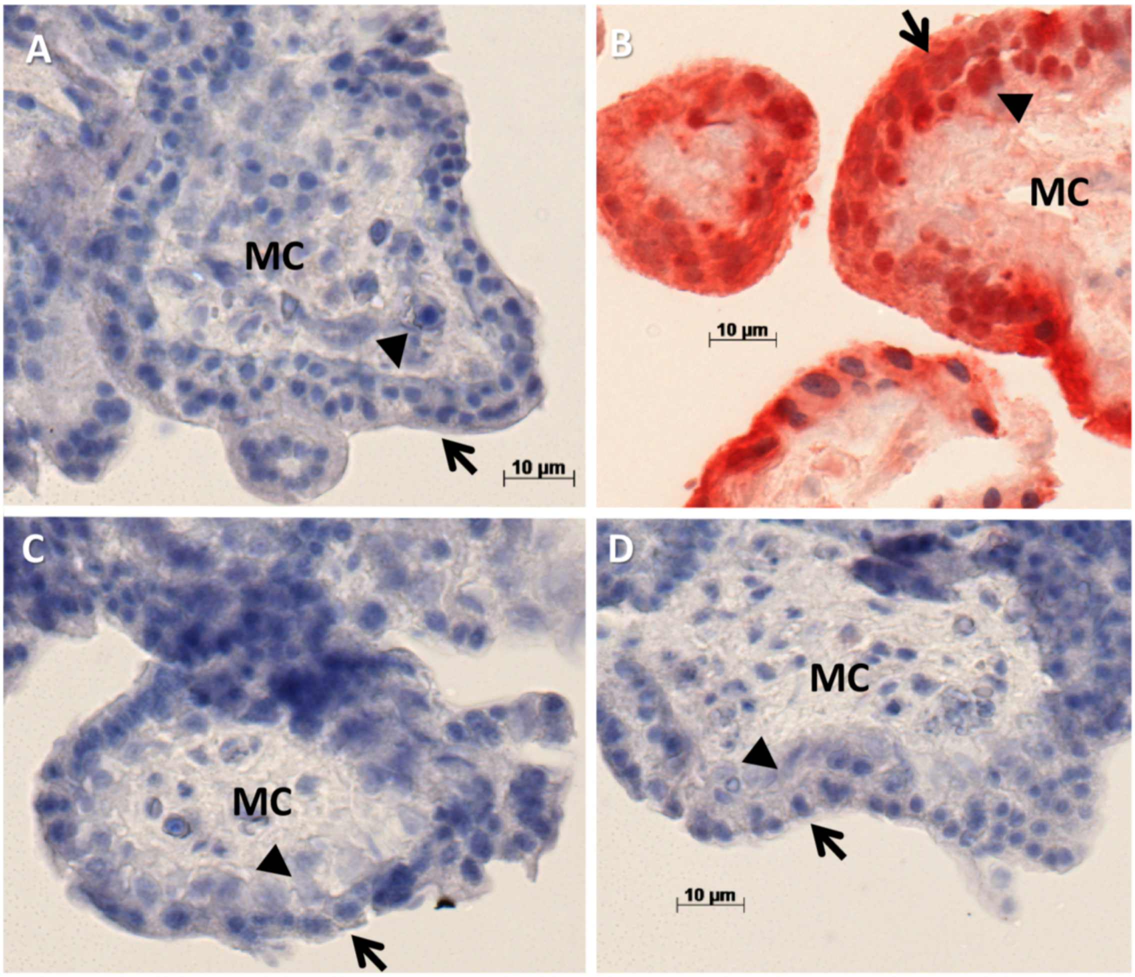

Sections of paraffin embedded first trimester placental explants that were exposed to 1 µM dPG-NPs (neutral, A,C;D; positively charged B), labeled with biotin, for 24h. Biotin was detected by a Streptavidin-Peroxidase–AEC detection system resulting in red-brown staining of NP deposits. Mayer's hemalum was used for tissue and nuclear counterstaining in blue. No signal for NP-accumulation could be detected in explants that were exposed to neutral dPGs (A,C,D) neither in the syncytium (black arrows) nor in the cytotrophoblast (black triangles).
There is also no signal indicating significant accumulation of neutral dPGs in the mesenchymal core (MC). In contrast, a very bright AEC-signal can be detected in the trophoblast layers, as well as in the MC of explants exposed to positively charged dPGs labelled with biotin (B).

Supplementary figure S4

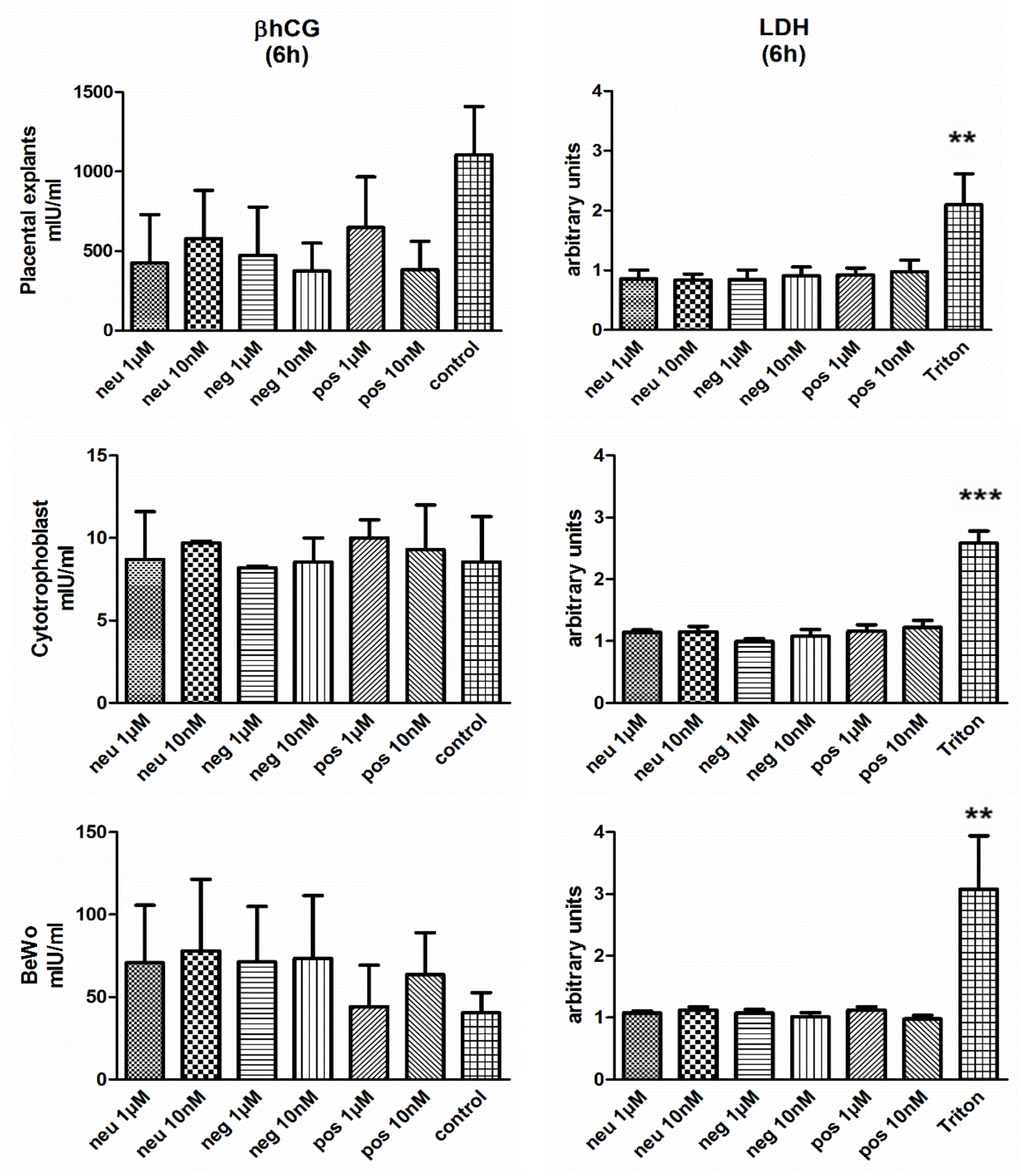

LDH -and hCG-levels in culture supernatant of placental explants, BeWo and primary cytotrophoblast cells, exposed to neutral (Neu), positively-(Pos), and negatively-charged (Neg) dPG-NPs at concentrations of 1 µM and 10 nM and to 1% Triton®X100 for 6h. LDH data are shown in relative units as ratio to control values, while hCG data are presented in mU mL-1. **(p<0.01) and ***(p<0.001) indicate statistically significant differences

Supplementary figure S5

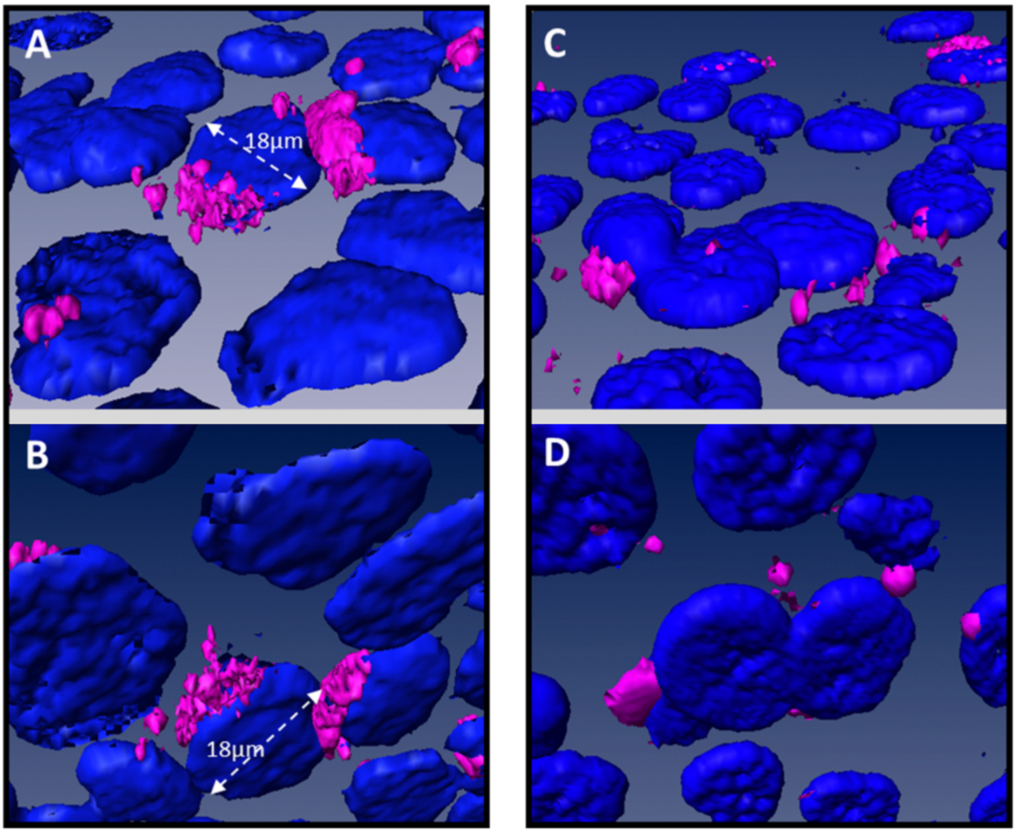

3D reconstructions of a BeWo cell- monolayer layer (A,B) and a primary trophoblast syncytium (C,D) of ~15µm height, containing Neg. dPG-NP deposits, from the LSM Z-stacks. Nuclei are blue and dPG-NP deposits are pink. A and C represent ~ 35° birds eye views on the layers, B and D ~35°views on the basis of the cell layers on the cover slip from below. Perinuclear dPG-NP deposits range from the basal to the apical parts of the nuclei, indicating intracellular location.

Supplementary figure S6

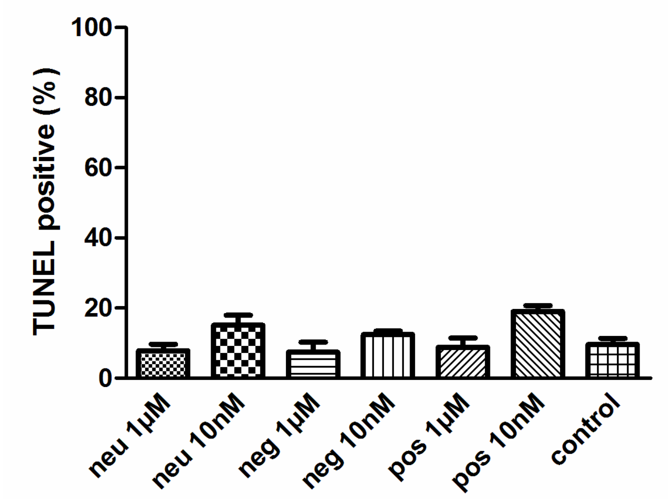

Percentage of TUNEL- positive nuclei in E-cadherine positive villous trophoblast, exposed to neutral (Neu), positively (Pos), and negatively charged (Neg) dPG-NPs for 24h at concentrations of 1 µM and 10 nM.

Supplementary figure S7

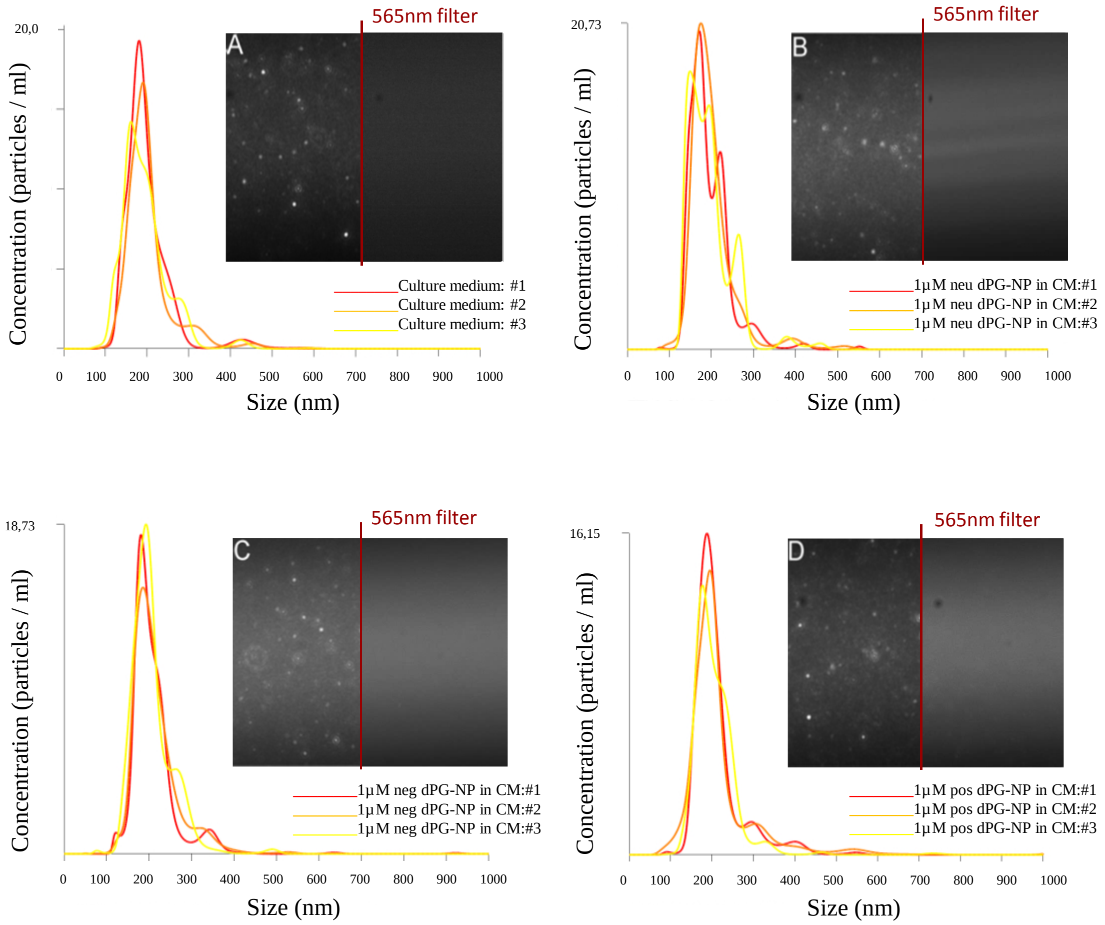

Results of nanoparticle tracking in culture medium (DMEM, 10% FCS) A, in culture medium (CM) containing neutral dPG-NPs (B), in CM containing negatively charged dPG-NPs (C), and in CM containing positively charged dPG-NPs (D). The concentration of the differently charged dPG-NPs in CM was 1 µM. The graphs show three calculations of the size distributions based on three measurements (red, orange and yellow lines). Microscopy images show examples of the laser-light scattering, used to calculate the NP-size. The right half of the images shows the filtered view, excluding wavelengths below 565 nm but allowing scattering of rhodamine B fluorescence labelled particles to pass through. The size of the most abundant serum proteins and of the dPG-NPs is below the detection limit of the method (30-40 nm), but obviously particles of a hydrodynamic diameter between ~50 and ~ 400 nm, can be detected in the CM.
Adding dPG-NPs does not significantly alter the size distribution pattern of these particles, irrespective of dPG-NP surface charge. In the 565 nm filter-view, the scattering of non-fluorescent particles is eliminated and no evidence for CM-induced aggregation of fluorescence-labeled NPs above the size detection limit of 30-40 nm can be observed.
